# Ligand B‐Factor Index: A Metric for Prioritizing Protein‐Ligand Complexes in Docking

**DOI:** 10.1002/minf.70010

**Published:** 2025-09-10

**Authors:** Liliana Halip, Cristian Neanu, Sorin Avram

**Affiliations:** ^1^ Department of Computational Chemistry “Coriolan Drăgulescu” Institute of Chemistry Timișoara Romanian Academy Timișoara Romania

**Keywords:** B‐factor, crystal‐structures, docking, scoring functions

## Abstract

Docking is a structure‐based cheminformatics tool broadly employed in early drug discovery. Based on the tridimensional structure of the protein target, docking is used to predict the binding interactions between the protein and a ligand, estimate the corresponding binding affinity, or perform virtual screenings (VSs) to identify new active compounds. This study introduces the ligand B‐factor index (LBI), a novel computational metric for prioritizing protein‐ligand complexes for docking. Unlike other metrics, LBI directly compares atomic displacements in the ligand and binding site. LBI is defined as the ratio of the median atomic B‐factor of the binding site to that of the bound ligand. Using the comparative assessment of scoring functions (CASF‐2016) dataset, we evaluated the effectiveness of LBI in guiding the selection of protein‐ligand complexes to enhance docking performance. Our results show a moderate correlation (Spearman *ρ* ~ 0.48) between LBI and the experimental binding affinities, outperforming several docking scoring functions. Additionally, LBI correlates with improved redocking success (root mean square deviation < 2 Å), underlying the significance of a ligand‐focused metric. While LBI outperforms other metrics such as the protein B‐factor index and resolution, its utility in VS docking remains to be further investigated. LBI is easy to compute, interpretable, applicable in structure‐based cheminformatics, and freely available for calculation at https://chembioinf.ro/tool‐bi‐computing.html.

## Graphical abstract

Ligand B‐factor index (LBI) is a computational metric for prioritizing protein‐ligand complexes for docking. LBI is defined as the ratio of the median atomic B‐factor of the binding site to that of the bound ligand, comparing atomic displacements in the ligand and binding site.

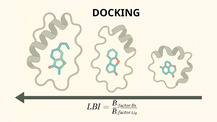

**Abbreviations**
DSFs: Docking Scoring Functions
EF: Enrichment Factor
eROCE: exponential Retrieval Operating Curve Enrichment
LBI: Ligand B‐factor Index
minBAG: minimum Binding Affinity Gap
pBA: log10(BindingAffinity[M])
PBI: Protein B‐factor Index
Res: Resolution of crystal structures

## 1 Introduction

Protein‐ligand docking is a critical cheminformatics technique used to predict the preferred orientation of a small‐ molecule (ligand) when bound to its protein target [1]. The docking process involves generating potential ligand‐binding poses within the protein binding pocket, followed by the computation of a scoring function that reflects the binding affinity based on interactions such as hydrogen bonding, hydrophobic effects, and electrostatic forces. Advances in scoring methods and hybrid models have enhanced the reliability of docking predictions, bridging computational insights with experimental validation [2].

The success of protein‐ligand docking predictions is intrinsically tied to the quality of crystal structures, which define the spatial and chemical landscape of the binding site. High‐resolution crystal structures with minimal conformational artifacts offer a reliable basis for identifying key binding interactions and optimizing docking algorithms. Conversely, poor‐quality structures may introduce errors such as missing residues, misoriented side chains, or unrealistic binding site geometries, leading to inaccurate docking results or false positives (FP)[3].

Various quantitative metrics prioritize crystal structures for docking, emphasizing structural accuracy and biological relevance. The crystal resolution [3], R‐factor (fit to experimental data), and free R‐factor (validation against unseen data) [4, 5] are some of the most widely known [6, 7]. Protein structure selection based solely on resolution has long been criticized because it measures the quantity of data collected and not the quality of the data or the fitted model [8], but continues to prevail across docking studies. More sophisticated quality metrics and workflows were proposed for Protein Data Bank (PDB) validation, e.g., the real space correlation coefficient and the real space R‐factor, real‐space difference density Z score, and real‐space observed density Z score scores as discussed by Tickle IJ [9]. Although these metrics were adopted by crystallographers, nonspecialists such as medical chemists and cheminformatics practitioners have not. Another metric, the diffraction‐precision index (DPI) [10], has been suggested for selecting PDB structures in docking studies [8], but computational complexity [11] and subsequent studies have indicated limited usability [12].

Recently, a new metric for PDB prioritization was proposed, based on the atomic B‐factor values available in the PDB files [12]. The B‐factor (also known as the Debye–Waller factor or temperature factor) reflects both vibration and static disorder in the crystal structures [13]. Indicating the relative vibrational motion of atoms in a protein, low value B‐factors can point toward well‐ordered sites, and contrary, atoms with high B‐factors might be part of the most flexible residues or regions [13, 14]. The B‐factor of the binding site index is calculated as the ratio of the average atomic B‐factors of the binding site and those of the protein [12]. This provides a normalized measure of how the flexibility (atomic motion) of the binding site compares to the rest of the protein. So far, the index has only been evaluated for pose prediction docking referencing AutoDock [15] results, and demonstrated superior capacity to prioritize PDB structures compared to other metrics such as DPI or R–R free [7, 12].

The introduction of new effective tools to prioritize protein‐ligand complexes for docking addresses a clear gap in the field. In this study, we propose a new B‐factor index, namely the ligand B‐factor index (LBI). The current study provides an extensive evaluation of LBI comprising multiple scoring functions employed in diverse docking tasks: binding affinity prediction, pose prediction, and virtual screening (VS). We hypothesize that LBI, by quantifying the relative atomic fluctuation of ligand and binding site, will outperform PBI and resolution (Res) in docking structure prioritization and can significantly enhance docking results.

## 2 Materials and Methods

### 2.1 Data Sets

The comparative assessment of scoring functions (CASF) benchmark set [16, 17] was downloaded (accessed April 20, 2024). CASF‐2016 was designed as a “scoring benchmark”, where the performance of scoring functions is measured decoupled from the docking process [17]. It comprises the 285 protein‐ligand PDB structures organized around 57 targets (each target with five ligands), crystal resolution, target assignment, experimental protein‐ligand binding affinities (Ki and Kd) converted to −log10(BindingAffinity[M]) (denoted for this article as pBA), and the docking results of 25 scoring functions. Considering that several of these scoring functions come with 1–3 implementations providing different outcomes, each of the docking scoring functions (DSFs) was treated separately, providing a total of 34 DSFs [17] to be investigated in the current study. The DSFs were compared in the following applications of docking: i) “ranking power” the ability to correctly rank the known ligands of a certain target protein by their binding affinities when the precise binding poses of those ligands are given (“scoring power”, also available in the CASF paper is very similar to ranking power and has been omitted); ii) “docking power”: the ability to identify the native ligand binding pose among computer‐generated decoys; and iii) “screening power” the ability to identify the true binders to a given target protein among a pool of random molecules [17]. The results obtained from the CASF‐2016 study were used to assess the capacity of B‐factor indices (BI, i.e., LBI and PBI) to lead the selection of PDB structure in docking.

### 2.2 Data Retrieval

Using package “bio3d” available in R statistical software platform [18] the protein‐ligand complexes from CASF‐2016 were downloaded as PDB files from RSCB PDB [6, 19]. The B‐factor values of ligand and the protein heavy atoms in the first configuration (if multiple were available) were retrieved.

### 2.3 Computation of B‐Factor Indices

Based on the atomic B‐factors of the heavy atoms, the LBI and PBI were computed as follows

(1) $$\mathrm{LBI}=\;\frac{BF_{\mathrm{BS}}}{BF_{L}}$$

(2) $$\mathrm{PBI}=\;\frac{BF_{\mathrm{BS}}}{BF_{P}}$$

where *BF*_BS_ is the median atomic B‐factor of the binding site, *BF*_P_ is the median atomic B‐factor in the protein, and *BF*_L_ is the median atomic B‐factor in the ligand. The median was preferred over the mean to reduce the influence of potential outliers (frequently encountered among atomic B‐factors), particularly in small‐molecule ligands. LBI and PBI were computed for several predefined radii of the binding site: 5, 10, 15, and 20 Å, as measured from the heavy atoms of the bound ligand. Thereby, we aim to explore potential variations in LBI and PBI performance as a function of the size of the binding site.

### 2.4 Data Sampling

PDB entries (protein‐ligand complexes) were sampled based on a predefined minimum binding affinity gap (minBAG). For example, if a minBAG of 0.5 ‐log(BindingAffinity[M]) was selected, in the list of protein‐ligand complexes ordered increasingly according to the binding affinities, the difference between two consecutive values should be ≥ 0.5. We employed the following algorithm: first, a random activity value was selected between 2 and 14 (reflecting the range of the available pBAs in the CASF‐2016 data), and a vector was built with increments of 0.5 toward both ends of the activity range. The closest activity values in the data set to each value in the vector were selected. From these values, we randomly sampled three‐quarters of the data points. This final step addressed the tendency to select (more frequently) the marginal activity points due to the normal distribution of activity values in the data sets (Supporting Information Figure S1). This sampling of the PDB entries was performed 1000 times, and mean, median, and confidence intervals were computed.

### 2.5 Performance Measures and Statistical Difference

#### 2.5.1 Correlation

The Spearman *ρ* correlation coefficient (Equation 3) was employed (using R statistical programing software) [18] to measure quantitatively the association between experimental binding affinities and BIs, Res, and the scores resulted from the redocking of the ligand into their cognate protein‐ligand complex computed with 34 scoring functions.

(3) $$\rho \;=1\;-\;\frac{6\;\sum \;d_{i}^{2}}{n(n^{2}-1)}$$

where *d* is the difference between the two ranks of each observation and *n* is the number of observations. A Spearman's *ρ* value of 0 indicates no correlation. Values of 1 and ‐1 indicate maximum positive and negative, respectively, rank correlation, i.e., the series which are compared have the same order, or the order is inverse, respectively. Several levels of correlation can be defined based on the *ρ* Spearman coefficient: “very weak” from 0.00 to 0.19, “weak” from 0.20 to 0.39, “moderate” from 0.40 to 0.59, “strong” from 0.60 to 0.79, and “very strong” from 0.80 to 1.0.

#### 2.5.2 VS

The VS capacity of the methods was measured using the metrics described in Table 1. The exponential receiver operating curve enrichment (eROCE‐ Equation (4)) [20] averages the exponential weights assigned to each true positive according to its corresponding FP rate. The parameter α adjusts the focus on the top instances. Here we used α = 32.19, meaning that actives ranked before the 5% FPs receive exponentially decreasing weights from 1 to 0.2 [20]. The eROCE parameter is a robust measure of the early enrichment of positive instances in binary classification tasks, particularly when the true positives are rare or when early correct identification of positives is critical such as in VS.

**TABLE 1:** Performance metric for VS.

Name	Functions^a^	Eq. No.
eROCE	$\text{eROCE}=\;\frac{1}{N_{A}}\sum_{i=1}^{N_{A}}e^{-\;\alpha \;\cdot \;{\mathrm{FPR}}_{i}},\;\alpha =32.19$	(4)
Enhancement factor at 1%, 5%, and 10%	$\mathrm{EF}\alpha =\;\frac{N_{A\alpha }\;}{N_{A}\;\cdot \;\alpha \;}\;,\;\alpha =\left\{1\%,\;5\%,\;10\%\right\}$	(5)

a $N_{A}$ ‐ number of actives (positives), $N_{\mathrm{A\alpha}}$ ‐ number of actives (positives) at α % of the data set, FPR ‐ false positive rate.

The enhancement factor (EFα; (Equation 5)) at 1%, 5%, and 10% of the data set quantifies the proportion of true positives captured in the top α% of ranked instances relative to the proportion expected by random chance. For example, at α = 1%, if EF1% = 1, the evaluated method performs no better than random chance. An EF1% > 1, indicates true positives are enriched more effectively than random chance, and higher values indicate better performance. If the EF1% < 1, the top 1% of predictions are worse than random chance. Successful VS was defined as eROCE > 0.1, which is equivalent to an EF1% of >5, EF5% of >3, and EF10% of >2 (as shown in Results).

The pairwise Wilcoxon nonparametric test [21] was employed to assess statistical significance between two groups of data. The test was performed using the function “pairwise.wilcox.test” available in package “stats” (version 3.6.2) in R statistical software platform [18].

## 3 Results

### 3.1 Spread in Binding Affinities Impacts Ranking Power

Cheminformatics methods capable of estimating the experimentally determined binding affinities of protein‐ligand complexes are essential for early drug discovery. A good correlation between the computer‐based score and experimental values can lead to a more cost‐efficient compound prioritization as drug candidates. This feature of the DSF was assessed in the CASF‐2016 paper through “ranking power”, i.e., the ability of DSFs to correctly rank the protein‐ligand complexes according to their binding affinities [17]. The authors used 57 targets, each with five available ligands (i.e., 5 x 57 = 285 total protein‐ligand complexes ‐ PDBs) and corresponding pBA values (−log(BindingAffinity[M]) ranging from 2 to 14 (see Figure S1 in Supporting Information). The ranking power of the 34 DSFs was described in terms of Spearman correlation (between the pBA and docking scores) for each protein target. Confidence intervals were computed for each DSF by sampling through the 57 correlation values corresponding to the protein targets. However, the correlation values computed based only on five complexes had uneven pBA gaps between the samples. For example, the pBA values available for the ligands of target 1 increase with a relative constant step of 2 log units as follows: 2.89, 5.66, 7.96, 9.00, and 11.09, resulting in a mean binding affinity gap of 2.05 ± 0.73. But, in the case of target 49, the pBA values increase with a mean of 0.5 ± 0.3 as follows: 6.10, 6.50, 7.41, 7.80, and 8.10. For the latter target, the small differences between the pBA of the ligands substantially increase the difficulty of the DSFs to achieve high‐ranking power compared to target 1. Such differences and the small number of ligands per target can heavily bias the results of the rank power evaluation and may limit generalizability.

To overcome variability in affinity gaps and to increase the sample size, we randomly selected from 285 protein‐ligand pairs (median of 6.5, mean of 6.49 ± 2.17, with 90% of the values ranging between 2.86 and 10.03) regardless of the targets and set minimum affinity gaps (minBAGs) between the samples (see Methods section ‐ sampling) of 0.05, 0.1, 0.5, and 1.0. Sampling 1000 times resulted in mean BAGs of 0.08, 0.14, 0.7, and 1.57, respectively (see Table S1 in Supporting Information). For each run, *ρ* the Spearman correlation coefficient was computed between the binding affinities and the docking scores, the LBIs, PBIs, and Res.

**FIGURE 1:**
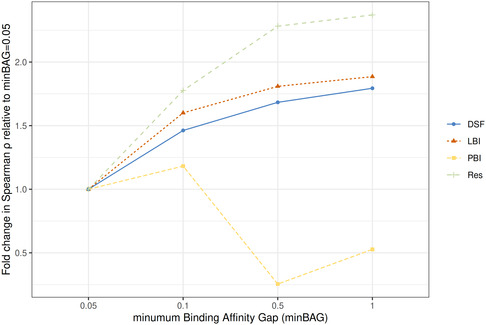
The ratios of the median Spearman correlations obtained for B‐factor indices (LBI and PBI), DSFs, and Res at various minBAGs relative to minBAGs of 0.05.

The median correlation at minBAG of 0.05 was considered as a reference to which results obtained at minBAG of 0.1, 0.5, and 1.0 were compared, as plotted in Figure 1. Except for PBI, for all parameters (LBI, DSFs, and Res), the ranking power increases with the increase of the gap between the pBAs. The correlation of Res increased > 2.5 times at minBAG of 0.5. The ranking power of LBI and DSFs varied similarly but with a ~ 1.5‐fold increase at 0.1, up to ~ 1.8 at minBAG of 1. In the case of PBI, the rank correlation dropped to around 0.5 as the spread in pBAs became larger (minBAG ≥ 0.5). The binding of the ligand only indirectly impacts the computation of PBI as reflected by the B‐factor of the binding site, which can explain why PBI is not influenced by the variations in binding affinities

For LBI, Res, and DFSs, the spread in binding affinities can significantly impact ranking power assessments. Although LBIs and DSFs seem to be affected in a similar manner these results emphasize the need for careful dataset curation in the evaluations of docking performance.

### 3.2 Ranking Power

The largest increase in ranking capacity was determined between minBAG of 0.05 and 0.1 (Figure 1). Hence, we further investigate the ranking power capabilities of LBI and PBI in comparison to DSFs and Res, using the results obtained at a minBAG of 0.1 (see Figure S2 and Table S2 in Supporting Information for results on different minBAGs).

**FIGURE 2:**
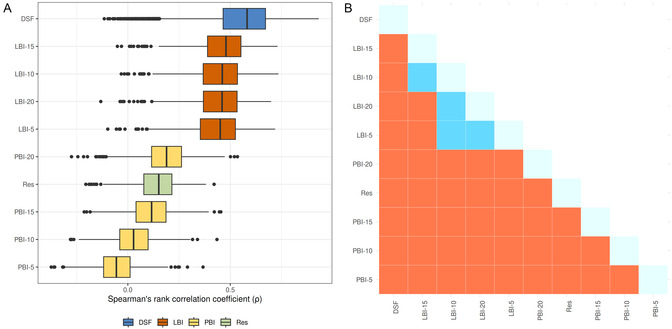
(A) Distribution of Spearman correlation values between the 285 experimental binding affinities (pBAs) and LBI, PBI, DSFs, and Res, based on 1000 resampling of protein‐ligand complexes drawn for a minimum minBAG of 0.1. LBI and PBI values were computed for binding sites of radius 5, 10, 15, and 20 Å around the ligand of the PDB complexes. (B) Significant differences in medians are colored red, i.e., *p* < 0.05, and blue otherwise, i.e., *p *≥ 0.05 (n.a. in light blue). For example, the correlation of pBAs with LBI‐15 is significantly higher than LBI‐20, LBI‐5, all PBIs, and Res (red), but not significantly different from LBI‐10 (blue).

Moderate correlation with the pBAs was observed for the aggregated DSFs, with a median of 0.58 (mean 0.56 ± 0.152) as shown in Figure 2A (individual DSF performances are shown in Figure S2 in Supporting Information). DSFs are closely followed by LBIs, also with moderate correlation, i.e., median of 0.48 and mean of 0.46 ± 0.126 (small differences in terms of radii ‐ Figure 2A). In the case of Res, we found low to very low correlation (median of 0.15). Finally, very poor or no correlation was observed in the case of PBI (medians between ‐0.06 for PBI‐5 and 0.19 for PBI‐20). The statistical significance test (Wilcoxon nonparametric test, *p* < 0.05) reveals that DSFs outperform LBIs, PBIs, and Res (Figure 2B). Although in comparison to LBIs, DSFs are more complex and require significantly more resources (time, costs and computational power), LBI‐15 performance is statistically superior to 7 DSFs: PMF@Sybyl, PMF04@DS, Goldscore@GOLD, LigScore1@DS, LondonG@MOE, GlideScore‐XP, and PMF@DS (Figure S3 in Supporting Information). These results suggest that LBI offers an efficient method for ranking protein‐ligand complexes in the PDB based on binding affinities, serving as a preliminary step before more complex computational analyses.

### 3.3 Pose Prediction

We next evaluated the utility LBI in the prioritization of PDBs for pose prediction. The RMSD values between the binding pose of the ligand in the complex (native binding pose) and the best‐scored binding pose selected by each scoring function were retrieved from CASF‐2016 and employed to compute the capacity of LBIs, PBIs, and Res to identify successful pose prediction docking, i.e., RMSD < 2 Å [17].

The capacity of a tool to prioritize PDBs that would result in successful pose prediction is highly dependent on the DSF employed. We can assume that for a given PDB, the more DSFs succeed in finding the native ligand pose (RMSD < 2 Å), the better the protein‐ligand complex is for pose prediction.

**FIGURE 3:**
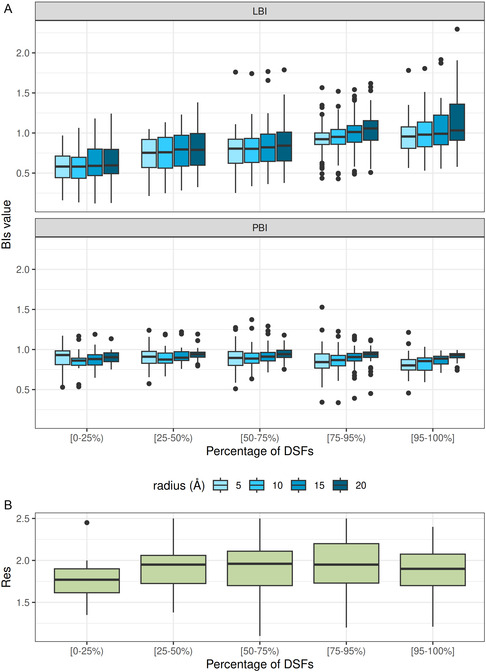
Distribution of B‐factor indexes ‐ (A) BIs and (B) Res values computed for PDBs grouped according to percentage of the DSFs achieving successful redocking of the native ligand (RMSD < 2 Å). LBI and PBI values were computed for binding sites of radius 5, 10, 15, and 20 Å around the ligand of the PDB complexes.

CASF‐2016 provides redocking results for 34 DSFs, which we used to group the 285 PDBs according to the percentages of DSFs that resulted in successful redockings: <25% DSFs ‐ 24 PDBs, 25–50% DSFs ‐ 47 PDBs, 50–75% DSFs ‐ 85 PDBs, 75–95% DSFs ‐ 103 PDBs, and > 95% DSFs ‐ 26 PDBs (Table S3 in Supporting Information). The distribution of the PDBs in the five groups was plotted against LBI, PBI, and Res as shown in Figure 3A,B.

In the case of LBI (Figure 3A), there is a clear trend indicating that the likelihood of a successful redocking achieved by a random DSF increases as the corresponding LBI value increases toward 1. The 24 PDB complexes (8%) for which less than 8 DSFs (< 25%) recognized the native ligand pose indicate LBI values ~ 0.6. In the 25–75% DSFs group (9 to 26 DSFs), successfully redocked the ligands of 129 complexes, with LBI values increased to ~ 0.80. For almost half of the PDBs (132; 46%) with a median LBI value of ~ 1, the majority of the DSFs (>75%, i.e., >27 of 34) were successful. These PDBs are more likely to result in RMSD < 2 Å redocking regardless of the DSF employed.

No significant differences were found between the LBI values as a function of the radius (Figure 3A), although in the > 75% DSFs group, a larger radius tends to increase the LBI values: LBI‐5 and LBI‐10 are very similar, i.e., median of 0.94 and 0.96, respectively, but LBI‐15 and LBI‐20 indicate slightly larger medians: 1.01 and 1.06, respectively. These results suggest that PDBs with LBI values ~ 1 should be prioritized for docking.

By increasing the radius, atomic displacements outside the close contact of ligands in the binding site are captured in the median B‐factor of the binding site component, but it does not significantly affect the LBI values (Figure 3A). For PDBs resulting in successful recognition of the native ligand conformation by > 75% of the DSFs, independent of the radius, the vast majority (75%) of the LBI values were bounded between 0.8 and 1.2 (Figure 3A), with medians ~ 1, i.e., 0.94 for LBI‐5, 0.96 for LBI‐10, 1.01 for LBI‐15, and 1.06 for LBI‐20.

PBI and Res appear to be relatively insensitive to the redocking results, as shown in Figure 3A,B and Table S4 in Supporting Information. Regardless of the DSFs percentage group, the PBI values remain relatively constant, with a median and mean of 0.90 (± 0.116). Moreover, the radius of the binding site does not affect the PBI performance (Figure 3A). The PDB structures for CASF‐216 data set indicate all relatively low Res, i.e., <2.5 Å. Thus, the selection of PDB from a set of low‐resolution (<2.5 Å) entries does not impact the docking power.

### 3.4 Virtual Screening

The ability of a DSF to identify the true binders of a protein target immersed in a pool of random compounds was computed and described in the CASF‐2016 paper as “screening power” (i.e., VS) [17]. In essence, each of the 57 protein targets was represented by five complexes. Thereby, five ligands per target were considered positives, and the ligands of the remaining 280 complexes were labeled as negatives.

**FIGURE 4:**
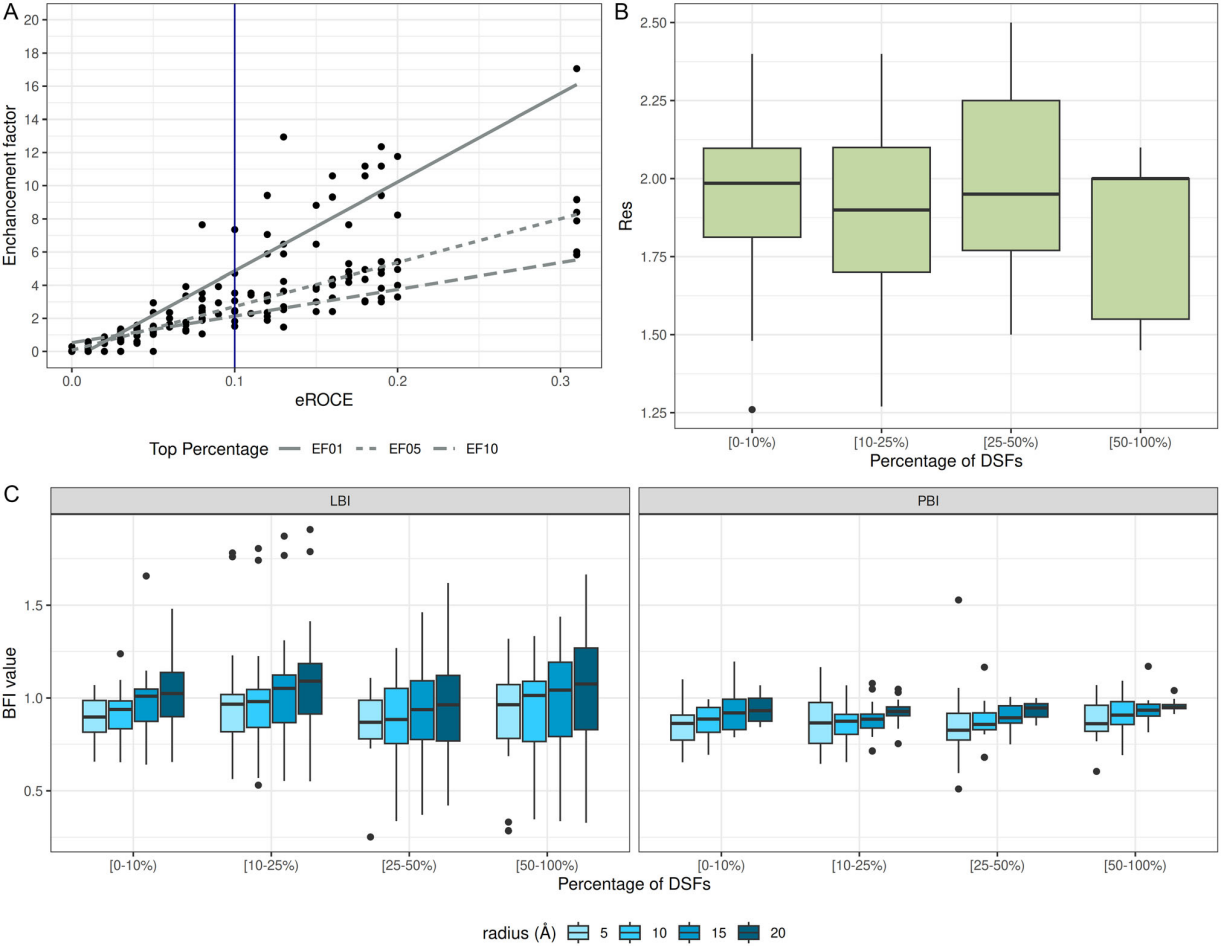
(A) The average eROCE (α = 32.19 ~ 5%) plotted against the average enhancement factor at the top 1, 5, 10% of the 54 docking targets used in the VS study, over the DSFs. (B) Distribution of Res and (C) B‐factor indices ‐ BIs, and values according to groups of targets for which successful VS was obtained by various percentages of DSFs. LBI and PBI values were computed for binding sites of radius 5, 10, 15, and 20 Å around the ligand of the PDB complexes.

We are interested in evaluating the capacity of B‐factor indices to guide the selection of crystal structures toward successful VSs for a majority of scoring functions. We define a cutoff for VS performance based on eROCE (Equation 4) [20, 22] to reference simultaneously enrichment factors computed for the top 1%, 5%, and 10%. For the purpose of this study, we consider successful VS to be reflected by an eROCE > 0.1. In terms of EF, it is equivalent to an EF1% of > 5, EF5% of > 3, and EF10% of > 2 (Figure 4A).

First, we found no Spearman correlation between eROCE and the three indices: LBI, PBI, and Res (1000 random sampling of 75% of the targets) (Table S5 in Supporting Information). Next, similar to the redocking experiment, the capacity of the indices to select protein‐ligand complexes for VS purposes was assessed by binning the results of the 57 targets according to the number of DSFs that resulted in successful VS (eROCE > 0.1). Up to 3 DSFs (i.e., <10%) achieved an eROCE > 0.1 in the case of 10 targets. Between 10% and 25% of the DFSs (4–8) were successfully applied for 17 targets. Also, VS performed on another 17 targets was successful for 9–17 DSFs. Finally, over 50% of the DSFs (>18) retrieved the required number of positives for 13 targets, suggesting a low agreement of DSF to enrich a common set of targets.

When plotted against Res (Figure 4B), LBI, and PBIs (Figure 4C), the results suggest a poor capacity to prioritize PDB structures for VS purposes. Variations in the binding site radius did not affect these results.

The VS results are not surprising because VS docking is more complex and subject not only to the atomic displacements of the ligand, binding site, and protein, as reflected by LBI and PBI (*vide infra*), but also to the docking algorithm, the type of target, reference PDB and the molecular properties of the chemical library. In the latter case, the VS design in CASF‐2016 considered large reference ligands with large differences in binding affinities (spreading over multiple log units) and low molecular similarity in comparison to the other ligands required to be retrieved for the same target (Figure S4 in Supporting Information). For more than 50% of the targets, on average, the reference ligand is at least 30% larger in terms of the number of atoms compared to the other ligands. Further, for 90% of the targets, on average, the reference ligand is at least 30% more active than the VS ligands. In terms of molecular similarity, in over 75% of the targets, the mean Tanimoto similarity between the reference ligand and the other actives is < 0.6 (Figure S4 in Supporting Information). Hence, the VS enrichment in CASF‐2016 relies on retrieving significantly less active and smaller ligands relative to the reference ligand determining the conformation of the binding site used in docking.

Although a direct correlation of BIs with VS results was not established, LBI can be indirectly employed to prioritize PDBs for VS docking based on the capacity to identify high‐affinity ligands and stable binding site conformations, as demonstrated above. Such properties can only increase the chances of finding actives binding the protein in a similar manner to the native ligand (potentially similar activity) and offer proper ground for more detailed investigations of the binding mode using more advanced techniques such as induced fit docking or molecular dynamics.

## 4 Discussions

### 4.1 Interpretation of LBI

LBI explicitly accounts for atomic movements in the ligand, a critical factor in estimating binding affinity toward a protein target. It compares the relative atomic displacement of the binding site to that of the ligand. Ligands with high atomic displacements (high B‐factors) may have weaker binding due to entropic penalties, while ligands with low atomic displacements (low B‐factors) may bind more tightly.

In the ranking power experiment, we observed a moderate positive correlation with binding affinities, indicating that higher LBI values are associated with stronger binding interactions. Successful pose prediction for the majority of the DSFs (>75%) was determined in PDBs for which an average LBI mean of 0.98 ± 0.196 was computed (Table S4 in Supporting Information and Figure 3A). These results indicate that PDBs with LBI values in range of 0.8–1.2 are more likely to result in successful docking for the radii 5 to 20 Å.

The selection of a radius to define the binding site might determine an optimal range for LBI. A smaller radius (e.g., 5 Å) will focus on directly interacting residues (first‐shell contacts) so that LBI will reflect differences in atomic‐level displacements between a few residues and the ligand. For a larger radius (e.g., 15–20 Å), LBI dilutes the B‐factor contrast between the binding site and the ligand, to a degree that captures possibly structural scaffolding of the binding site or its vicinity up to the entire protein, depending on the size of the protein and of the ligand.

In PDBs with low LBI (e.g., < 0.8), we might encounter a “rigid” binding site relative to a “movable” ligand, a less optimized binding interaction, due to weak or transient binding, or a ligand with high internal motion even when bound. This might explain the lower binding affinities for such protein‐ligand complexes. In the case of LBI ~ 1, the binding site and ligand have similar atomic displacements, suggesting a balanced interaction where neither is significantly more “dynamic”. This balance between flexibility (atomic displacement) and stability may favor strong and specific binding, explaining the higher success rate in the redocking experiments for PDBs associated with LBI values between 0.8 and 1.2. In high LBI (e.g., >1.2) crystal complexes, a flexible binding site might adapt to different ligands. Although it might be interpreted also as weaker binding due to increased conformational entropy, our results suggest in general higher binding affinities and accurate pose predictions.

### 4.2 Web Server

LBI and PBI can be computed fast and free of charge using the BI‐computing tool available on https://chembioinf.ro/tool‐bi‐computing.html. The platform provides an intuitive interface to query and retrieve PDB files. Interactive protein visualization (integrated with NGLViewerR [23]) enables users to explore the 3D structure of proteins and their ligand‐binding sites. The selection of the desired chain enables ligand selection (if available) and binding site definition based on a desired radius (Å). Based on the user selections, LBI and PBI are computed and printed along with supplementary parameters to assess the fit of the crystal structures for docking: number of atoms in the ligand and binding site, Res, R‐free, R, and DPI. Moreover, a graphical representation of the atomic B‐factors can help the identification of outliers in the protein, binding site, and ligand. The website was developed in the “shiny” [24] framework in the R programming language [18].

### 4.3 Use Case: B‐Factor‐Based Binding Site Analysis with the BI Computing Tool

Users start by retrieving a PDB entry using a 4‐character PDB code. Once the code is submitted, the determination method is displayed. If the structure was resolved using a method that does not provide B‐factor values (e.g., 2jnc with NMR), calculations cannot proceed. This information is also accessible through the info icon located next to the determination method.

**FIGURE 5:**
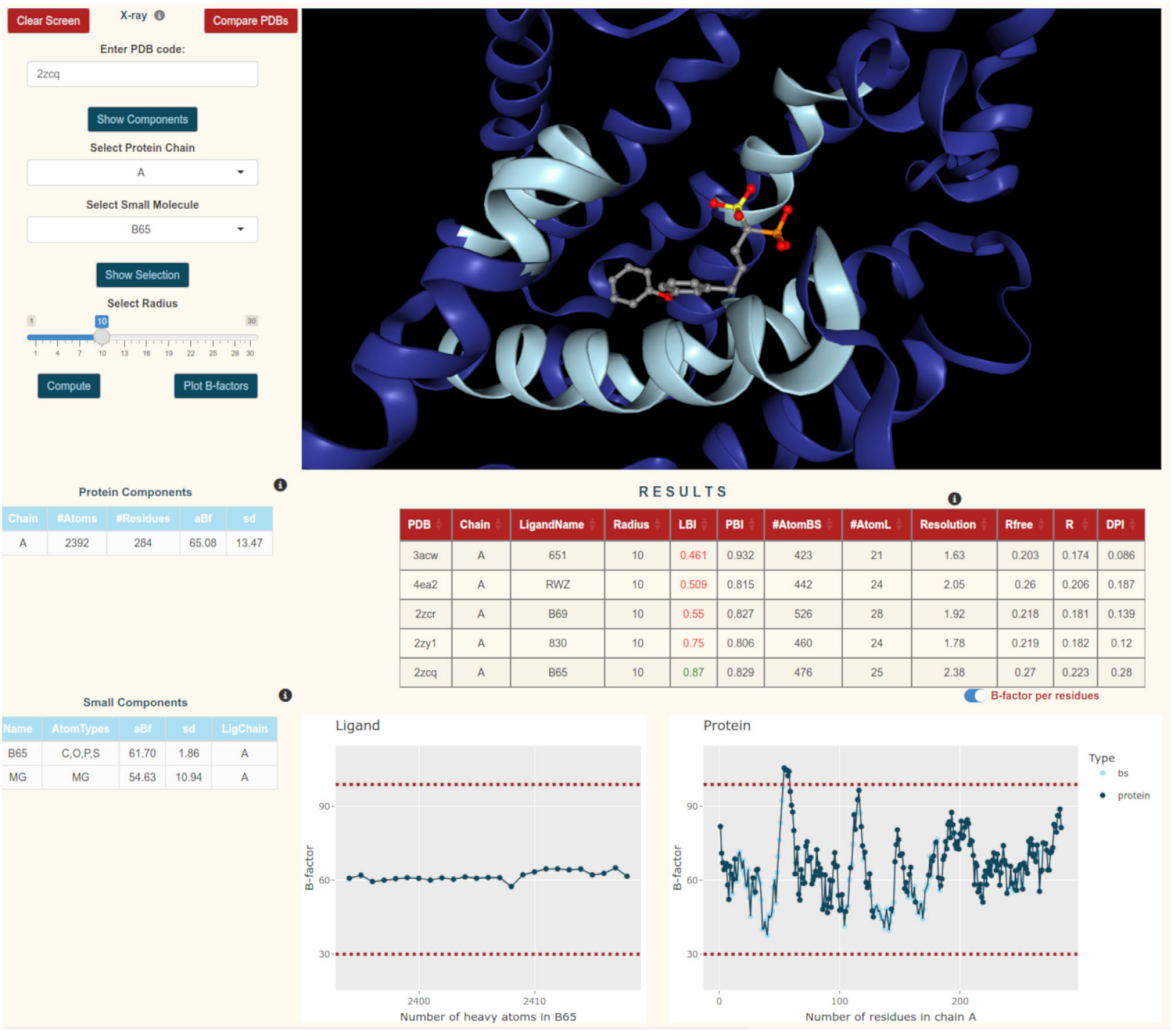
Example of LBI computation using BI‐computing tool (https://chembioinf.ro/tool‐bi‐computing.html).

As an example, we use the online BI‐computing tool to analyze the X‐ray crystal structure of a protein structure included in the CASF dataset, i.e., carotenoid dehydrosqualene synthase from *Staphylococcus aureus*. By entering the PDB code *3acw*, the PDB viewer displays the protein chain, its bound ligand, and water molecules. Upon loading the structure, the results table initially displays only basic metadata from the PDB file, such as resolution, R‐free, R‐work, and calculated DPI. Additional computed values are generated after the Compute button is pressed. By selecting chain A and activating the show selection option, the viewer isolates chain A and the ligand, automatically zooming in on the binding site, which is highlighted in light blue. The default binding site radius is set to 10 Å and is adjustable using the radius slider. Once the selections are made, pressing the Compute button triggers the calculation of ligand‐binding metrics. For chain A at a 10 Å radius, the computed LBI is 0.461, a value which falls outside the 0.8–1.2 range (see above). By clicking the compare PDBs button, a second PDB entry, such as *4ea2*, can be loaded while retaining the previous results, although they are temporarily not visible until a new structure appears in the viewer. For the newly loaded structure, the small‐molecule components table indicates three small molecules associated with the protein chain. The bound ligand can be identified by examining both the viewer window and the small‐molecule components table below. In this case, we identified and selected the ligand RWZ. The computed LBI in this example is 0.509, still below the 0.8 threshold. Repeating the selection and computation process for the other CASF structures of the same protein‐target (*2zcr*, *2yr1*, and *2czq*), more entries will be added to the results table, enabling direct comparison of different structures and/or binding site definitions. With a LBI of 0.87, *2czq* is the crystal structure recommended for docking (Figure 5).

In addition to numerical assessments, the BI‐computing tool also supports visual exploration of flexibility within the binding site. Selecting the plot B‐factor button generates B‐factor plots for both protein and ligand atoms. These plots highlight residues within the binding site and include statistical features such as the first and third quartiles, offering deeper insight into structural variability and mobility.

To manage the interface, the clear screen button removes all data and resets the app. In contrast, the compare PDBs feature retains all existing results, supporting side‐by‐side analysis across multiple structures of the same protein target.

The integration of these features into a single platform streamlines the workflow for researchers, eliminating the need for multiple tools or software to help decision‐making in PDB selection for structure‐based approaches. Due to a user‐friendly interface, with dynamic visualizations and real‐time updates that enhance data interpretation, the website serves as a valuable resource for structural biologists and computational chemists seeking to evaluate the dynamic properties of protein‐ligand interactions.

## 5 Limitations

One should keep in mind that B‐factor values reflect not only local movements but also crystal defects, large‐scale disorder, diffraction data quality, etc. [25], that can affect B‐factor indices values, particularly with smaller binding sites and ligands. Poorly resolved ligands may have unreliable B‐factors, leading to noisy or misleading LBI values. However, these are inherent experimental errors that should not impair the use of the B‐factor as a tool in cheminformatics. To help identify atomic B‐factors with abnormal values, we implemented the B‐factor plots as described above (Figure 5). Moreover, while B‐factors conflate thermal motion and disorder, our median‐based approach reduces sensitivity to outliers. B‐factors are influenced by numerous other experimental conditions (resolution, temperature, refinement, etc.) impairing direct comparisons between multiple PDB structures. But, in LBI and PBI, both the numerator and denominator are derived from the same crystal structure, subject to the same experimental biases. This internal normalization cancels or partially normalizes global scaling effects such as systematic inflation or deflation of B‐factors due to refinement strategy or resolution, making BIs more robust than comparing raw B‐factors between different PDBs.

The study is also limited to the size, design and results of CASF‐2016. Although CASF‐2016 protein‐ligand complexes may not fully represent the diversity of protein‐ligand interactions encountered in real‐world drug discovery, these are carefully selected protein‐ligand complexes with binding affinities spread over multiple log units for the majority of the ligands. Given the large number of DSFs compared in multiple docking tasks, the CASF‐2016 remains the most compelling and well‐rounded dataset for performance assessment in docking [26].

## 6 Future Directions

So far, we have focused on the potential of LBI to prioritize crystal‐structures of protein‐ligand complexes for docking based on a consistent number of scoring functions. Although we were able to demonstrate the utility of LBI in power ranking and pose prediction, its application in VS docking remains to be explored in future studies, using other datasets designed for this particular assessment, such as DUD‐E [27].

Our results encourage expanding the scope and applicability of LBI in drug discovery. For example, in lead optimization, LBI could guide the selection of ligand modifications to enhance binding affinity. By analyzing the LBI values of existing protein‐ligand complexes, medicinal chemists could identify structural features that contribute to favorable flexibility profiles and incorporate these insights into the design of new compounds. Similarly, in fragment‐based drug discovery (FBDD), where small, flexible fragments are screened for binding to a target protein, LBI can be employed to prioritize fragments that indicate a good balance of flexibility and rigidity (atomic displacements), increasing the likelihood of identifying fragments that can be optimized into high‐affinity binders. Such an approach could streamline the fragment screening process and improve the efficiency of FBDD campaigns.

LBI can not only improve the selection of PDBs for training modern scoring functions based on machine learning, but can also be integrated into such models to develop more robust scoring functions. This might be a promising lead since several methodologies successfully employed B‐factors integration into scoring functions to increase accuracy in protein‐ligand binding affinity prediction [28].

## 7 Conclusions

This study introduces and evaluates LBI as a novel metric for prioritizing protein‐ligand complexes in docking (binding affinity ranking, pose prediction, and VS), providing a comprehensive view of its utility. LBI integrates atomic B‐factors into a simple, interpretable metric that can be widely employed given the widespread availability of B‐factor data in PDB files, with implications for improving cheminformatics tools used in early‐stage drug discovery. The use of the CASF‐2016 benchmark set ensures that the results are comparable to existing DSFs and future developments in the field.

Key findings indicate the spread in binding affinities as a significant factor in performance evaluation of DSFs. A larger affinity gap between ligands within a given target improves ranking performance, particularly for LBI and DSFs, which exhibited moderate correlations with experimental binding affinities. Notably, LBI emerged as a useful descriptor, showing competitive ranking power to DSFs and even surpassing some in performance.

Regarding docking pose prediction, LBI was found to be a meaningful predictor of successful redocking, with LBI values ~ 1 correlating with improved pose selection by DSFs. This relationship suggests that atomic displacements in the ligand play a crucial role in docking accuracy.

Both properties, i.e., correlation with binding affinity and with pose prediction accuracy, indicate that LBI can serve for PDB selection in VS. Although BIs could not be correlated directly with the retrieval of active compounds, as designed in the current study, PDB structures prioritized with LBI tend to contain the most active ligand available (potentially increasing the retrieval of similarly active compounds in VS) and provide high accuracy in the analysis of the interactions between ligands and the protein.

Overall, based on the current results, we recommend prioritization of PDB structures showing LBI values between 0.8 and 1.2 for docking purposes.

PBI and Res did not exhibit significant correlations with successful docking outcomes, indicating that these parameters alone may not be reliable predictors in docking assessments.

LBI and PBI together with several other parameters and visual representations of PDB structures are freely available at https://chembioinf.ro/tool‐bi‐computing.html.

In a broader context, this study contributes to the ongoing refinement of cheminformatics tools applied to drug discovery. While traditional DSFs remain central to structure‐based VS, incorporating B‐factor‐based indices such as LBI can enhance the prioritization of protein‐ligand complexes with favorable binding characteristics. Future research should explore applications of LBI in machine learning‐driven scoring functions and expand testing to larger datasets.

## Supporting Information

**Supporting Fig. S1:** Histogram of pBA values (‐log(BindingAffinity[M])) of the 285 protein‐ligand complexes in CASF2016 data set. **Supporting Fig. S2:** Distribution of Spearman correlation values between BFIs, Res, and DSFs against experimental binding affinities (pBA) over 1000 resampling of protein‐ligand complexes based on 4 threshold values defining minBAGs of 0.05, 0.1, 0.5, and 1. **Supporting Fig. S3:** Boxplot showing the statistical difference (Wilcoxon nonparametric test, alpha ” indicates that the row instance (i.e., LBI15, LBI‐10, LBI‐20, and LBI‐5) surpassed the instance in the column. For example, at minBAG of 0.1, the Spearman correlation between pBAs and LBI‐15 is statistically superior (“>”) to PMF@Sybyl, PMF04@DS, Goldscore@GOLD, LigScore1@DS, LondonG@MOE, GlideScore‐XP, and PMF@DS but statistically inferior to the other DSFs. **Supporting Fig. S4:** Histograms describing the binding affinity (A), number of atoms (B), and Tanimoto distance (C) of the ligand in the reference PDB (i.e., used for VS docking) relative to the other ligands of the same target. **Supporting Table S1:** A summary of the 1000 samplings obtained by setting a minimum activity gap (minBAG) of 0.05, 0.1, 0.5, and 1.0. **Supporting Table S2:** A summary of the ranking power results at different minBAGs. **Supporting Table S3:** Number of CASF2018 protein‐ligand complexes (PDBs) in groups defined by intervals of percentages of docking scoring functions (DSFs) which resulted in successful redocking compared to the native ligand pose (RMSD < 2Å). **Supporting Table S4:** Median (mean ± standard deviation) of LBI, PBI, Res, corresponding to the protein‐ligand complexes (PDBs) for which successful redocking of the native ligand pose (RMSD < 2 Å) was computed by various numbers of DSFs groups in 6 percentage intervals. **Supporting Table S5:** Mean Spearman correlation (over 1000 random sampling of 75% of the 57 targets) between mean eROCE and LBI, PBI, and Res.

## Author Contributions

**Liliana Halip:** formal analysis and investigation, writing – original draft preparation. **Cristian Neanu:** formal analysis and investigation. **Sorin Avram:** conceptualization, formal analysis and investigation, writing – original draft preparation, writing – review and editing.

## Conflict of Interest

The authors declare no conflicts of interest.

## Supporting information

Supplementary Material

## Data Availability

Research data not shared.
