# Supplementary material for: Ligand B‐Factor Index: A Metric for Prioritizing Protein‐Ligand Complexes in Docking
Source: Mol Inform. 2025 Sep 10;44(9):e202500127. doi: 10.1002/minf.70010 (PMC12423484; doi:10.1002/minf.70010)
Supplement: Supplementary file 1 — Supplementary Material [file MINF-44-e202500127-s001.pdf]

Supporting information

Ligand B-Factor Index (LBI): A Metric for Prioritizing Protein-Ligand Complexes in Docking

Liliana Halip, Cristian Neanu, Sorin Avram*

Department of Computational Chemistry, Institute of Chemistry Timisoara, Romanian Academy, 24th Mihai Viteazu Avenue, Timișoara, 300223, Romania

Table S1. A summary of the 1000 samplings obtained by setting a minimum activity gap (minBAG) of 0.05, 0.1, 0.5, and 1.0.

Table S2. A summary of the ranking power results at different minBAGs

Table S3. Number of CASF2018 protein-ligand complexes (PDBs) in groups defined by intervals of percentages of Docking Scoring Functions (DSFs) which resulted in successful redocking compared to the native ligand pose (RMSD < 2Å)

Table S4. Median (mean ± standard deviation) of LBI, PBI, Res, corresponding to the protein-ligand complexes (PDBs) for which successful redocking of the native ligand pose (RMSD < 2 Å) was computed by various numbers of DSFs groups in 6 percentage intervals.

Table S5. Mean Spearman correlation (over 1000 random sampling of 75% of the 57 targets) between mean eROCE and LBI, PBI, and Res

Figure S1. Histogram of pBA values (-log(BindingAffinity[M])) of the 285 protein-ligand complexes in CASF-2016 data set

Figure S2. Distribution of Spearman correlation values between BFIs, Res, and DSFs against experimental binding affinities (pBA) over 1000 resampling of protein-ligand complexes based on 4 threshold values defining minBAGs of 0.05, 0.1, 0.5, and 1.

Figure S3. Boxplot showing the statistical difference (Wilcoxon non-parametric test, alpha < 0.05 - red, otherwise blue) in the distributions of Spearman rho computed between LBI and scoring functions. The results are shown for different activity gaps (minBAG): 0.05, 0.1, 0.5, and 1.0. The plus sign ">" indicates that the row instance (i.e., LBI-15, LBI-10, LBI-20, and LBI-5) surpassed the instance in the column. For example, at minBAG of 0.1, the Spearman correlation between pBAs and LBI-15 is statistically superior (">") to PMF@Sybyl, PMF04@DS, Goldscore@GOLD, LigScore1@DS, LondonG@MOE, GlideScore-XP, and PMF@DS but statistically inferior to the other DSFs.

Figure S4. Histograms describing the binding affinity (A), number of atoms (B), and Tanimoto distance (C) of the ligand in the reference PDB (i.e., used for VS docking) relative to the other ligands of the same target.

Table S1. A summary of the 1000 samplings obtained by setting a minimum activity gap (minBAG) of 0.05, 0.1, 0.5, and 1.0.

minBAG	Mean BAGs	Number of complexes sampled per run		
		mean	min	max
0.05	0.08	59	16	73
0.10	0.14	57	33	67
0.50	0.7	14	13	15
1.00	1.57	6	6	6

Table S2. A summary of the ranking power results at different minBAGs

Class	Type	minBAG: 0.05	minBAG: 0.10	minBAG: 0.50	minBAG: 1.00
LBI	LBI-5	0.28(0.28±0.147)	0.45(0.43±0.129)	0.48(0.45±0.213)	0.54(0.43±0.396)
	LBI-10	0.29(0.28±0.152)	0.46(0.45±0.129)	0.53(0.51±0.199)	0.54(0.47±0.371)
	LBI-15	0.29(0.29±0.148)	0.48(0.46±0.126)	0.54(0.53±0.188)	0.54(0.48±0.353)
	LBI-20	0.29(0.28±0.157)	0.46(0.44±0.128)	0.52(0.49±0.198)	0.54(0.45±0.355)
PBI	PBI-5	-0.03(-0.02±0.1)	-0.06(-0.05±0.101)	-0.15(-0.14±0.243)	-0.13(-0.13±0.403)
	PBI-10	0.04(0.03±0.109)	0.03(0.03±0.106)	-0.01(-0.01±0.248)	-0.03(-0.02±0.403)
	PBI-15	0.09(0.08±0.123)	0.12(0.11±0.109)	0.07(0.09±0.242)	0.06(0.06±0.387)
	PBI-20	0.14(0.12±0.136)	0.19(0.19±0.115)	0.13(0.14±0.219)	0.14(0.14±0.388)
Other	Resolution (Res)	0.09(0.09±0.119)	0.15(0.14±0.101)	0.19(0.19±0.208)	0.2(0.21±0.372)
	Docking Scoring Function (DSF)	0.4(0.39±0.163)	0.58(0.56±0.152)	0.67(0.63±0.198)	0.71(0.62±0.301)

Table S3. Number of CASF2018 protein-ligand complexes (PDBs) in groups defined by intervals of percentages of Docking Scoring Functions (DSFs) which resulted in successful redocking compared to the native ligand pose (RMSD < 2Å)

Percentage of Docking Scoring Functions	[0-25%)	[25-50%)	[50-75%)	[75-95%)	[95-100%]
Number of Docking Scoring Functions	0-8	9-16	17-25	26-31	32-34
Number of Protein-Ligand Complexes (PDBs)	24	47	85	103	26

Table S4. Median (mean ± standard deviation) of LBI, PBI, Res, corresponding to the protein-ligand complexes (PDBs) for which successful redocking of the native ligand pose (RMSD < 2 Å) was computed by various numbers of DSFs groups in 6 percentage intervals

Percentage of DSFs	LBI	PBI	Res
[0-25%)	0.58(0.6±0.252)	0.89(0.88±0.126)	1.77(1.75±0.231)
[25-50%)	0.78(0.77±0.241)	0.91(0.91±0.103)	1.95(1.89±0.256)
[50-75%)	0.82(0.87±0.527)	0.91(0.91±0.113)	1.96(1.91±0.345)
[75-95%)	0.98(0.98±0.196)	0.89(0.89±0.119)	1.95(1.95±0.309)
[95-100%]	0.99(1.04±0.33)	0.87(0.86±0.111)	1.9(1.87±0.308)

Table S5. Mean Spearman correlation (over 1000 random sampling of 75% of the 57 targets) between mean eROCE and LBI, PBI, and Res

Mean eROCE@	LBI- 5	LBI-10	LBI-15	LBI-20	PBI-5	PBI-10	PBI-15	PBI-20	Res
5% FPs	0.02	0.04	0	-0.01	-0.01	0.05	0.09	0.16	-0.02

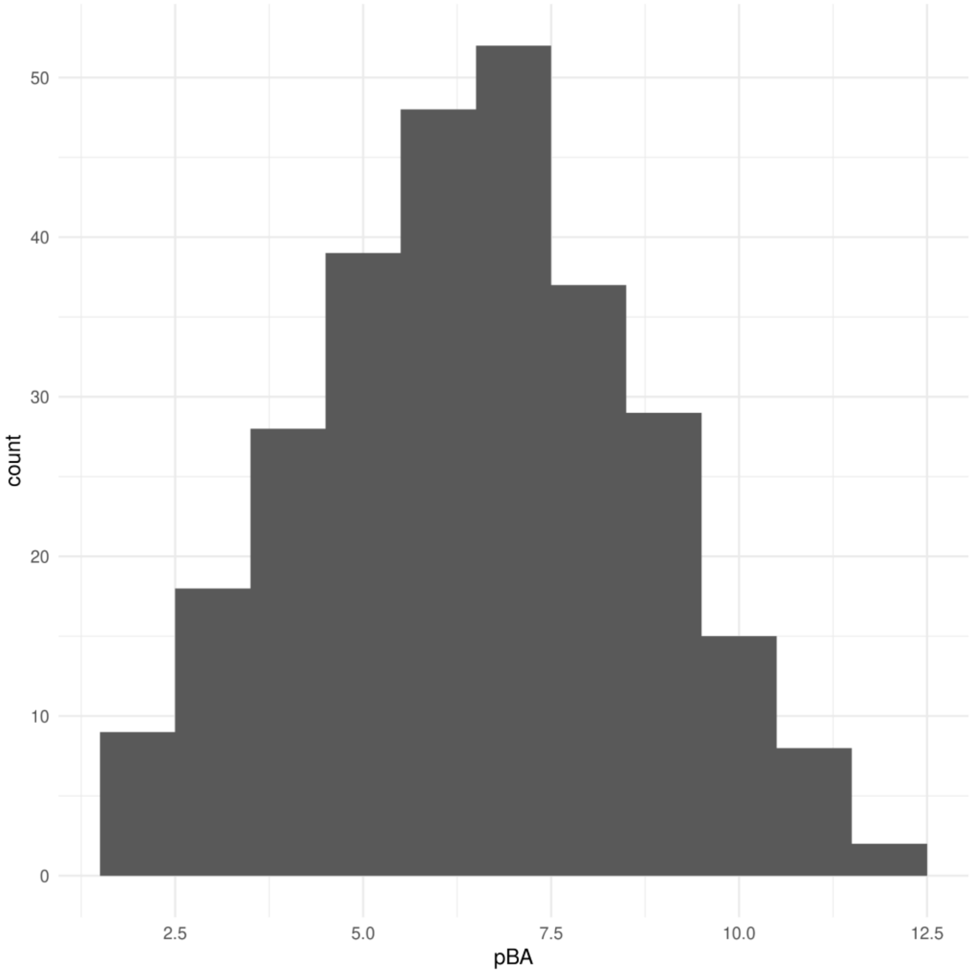

Figure S1. Histogram of pBA values (-log(BindingAffinity[M])) of the 285 protein-ligand complexes in CASF-2016 data set

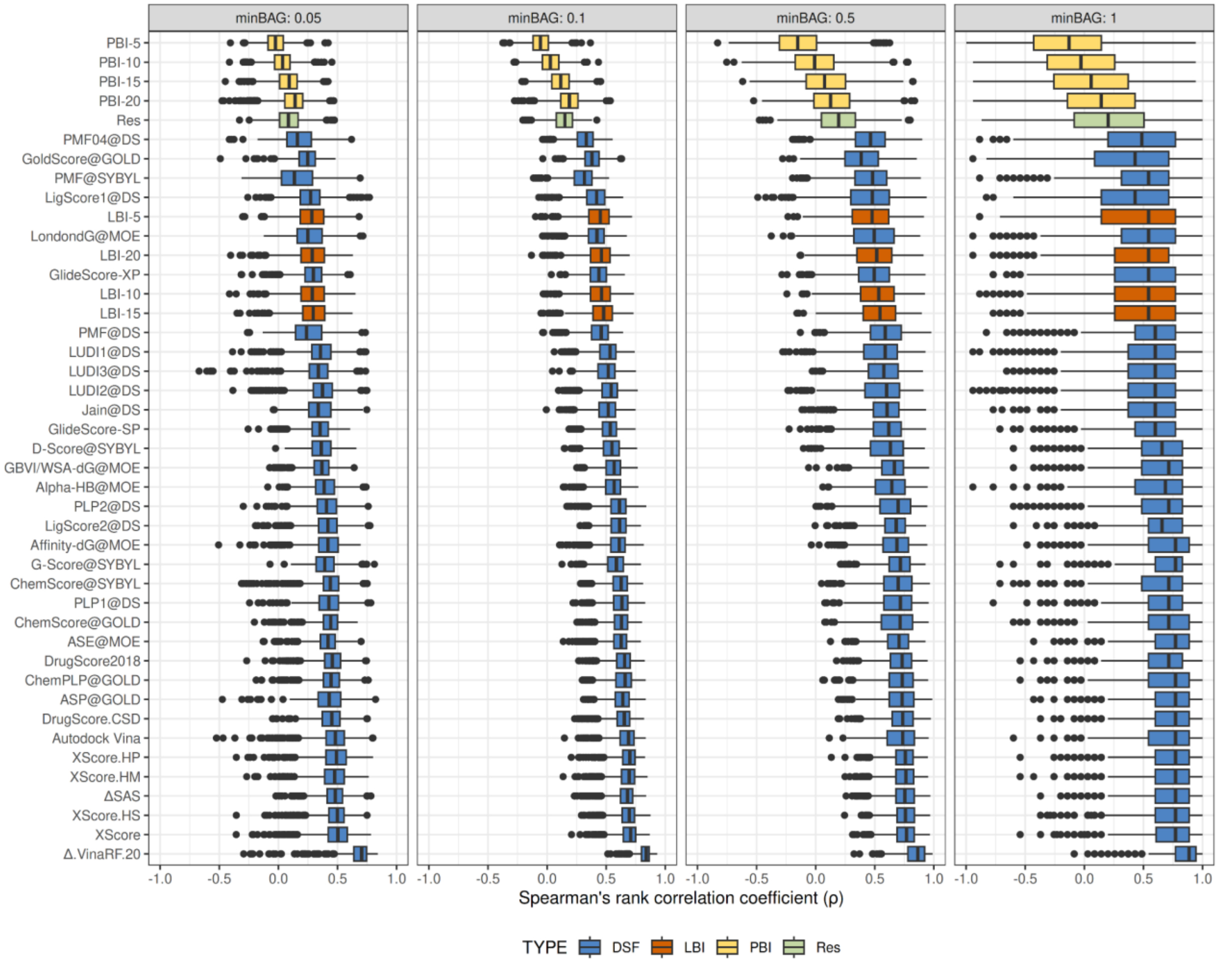

Figure S2. Distribution of Spearman correlation values between BFIs, Res, and DSFs against experimental binding affinities (pBA) over 1000 resampling of protein-ligand complexes based on 4 threshold values defining minBAGs of 0.05, 0.1, 0.5, and 1

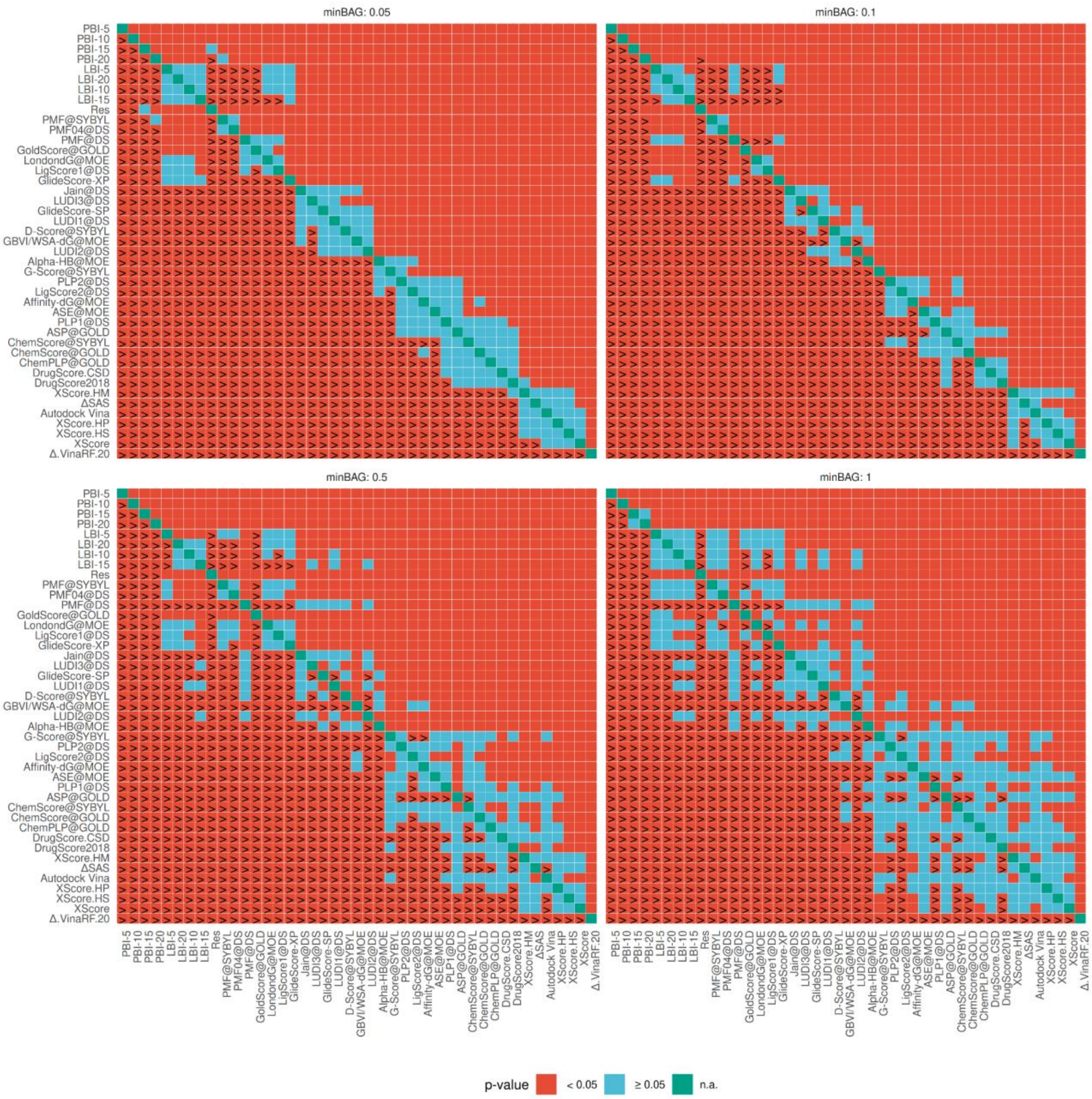

Figure S3. Boxplot showing the statistical difference (Wilcoxon non-parametric test, alpha < 0.05 - red, otherwise blue) in the distributions of Spearman rho computed between LBI and scoring functions. The results are shown for different activity gaps (minBAG): 0.05, 0.1, 0.5, and 1.0. The plus sign ">" indicates that the row instance (i.e., LBI-15, LBI-10, LBI-20, and LBI-5) surpassed the instance in the column. For example, at minBAG of 0.1, the Spearman correlation between pBAs and LBI-15 is statistically superior (">") to PMF@Sybyl, PMF04@DS, Goldscore@GOLD, LigScore1@DS, LondonG@MOE, GlideScore-XP, and PMF@DS but statistically inferior to the other DSFs.

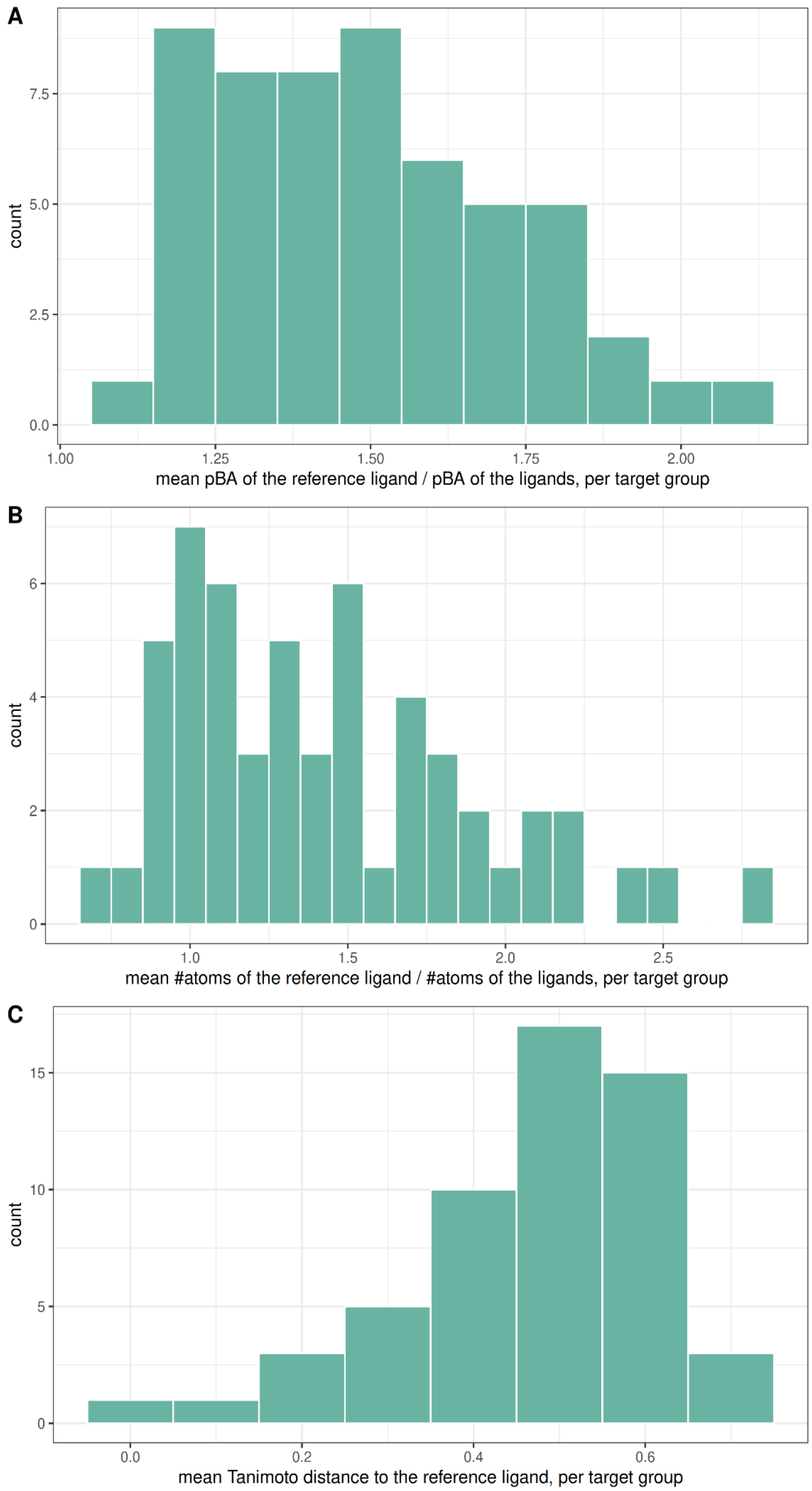

Figure S4. Histograms describing the binding affinity (A), number of atoms (B), and Tanimoto distance (C) of the ligand in the reference PDB (i.e., used for VS docking) relative to the other ligands of the same target.
